# Mindfulness Training: Can It Create Superheroes?

**DOI:** 10.3389/fpsyg.2019.00613

**Published:** 2019-03-26

**Authors:** Patrick Jones

**Affiliations:** School of Psychology and Exercise Science, Murdoch University, Perth, WA, Australia

**Keywords:** mindfulness, heroism, superhero, superpowers, human potential, meditation, enlightenment, Buddhism

## Abstract

With the emergence of the science of heroism there now exists both theoretical and empirical literature on the characteristics of our everyday hero. We seek to expand this inquiry and ask what could be the causes and conditions of a superhero. To address this we investigate the origins of mindfulness, Buddhist psychology and the assertion that its practitioners who have attained expertise in mindfulness practices can develop supernormal capabilities. Examining first their foundational eight “jhana” states (levels of attention) and the six consequent “abhinnas” (siddhis or special abilities) that arise from such mental mastery, we then explore any evidence that mindfulness practices have unfolded the supernormal potential of its practitioners. We found a growing base of empirical literature suggesting some practitioners exhibit indicators of enhanced functioning including elevated physical health and resistance to disease, increased immunity to aging and improved cognitive processing, greater resilience and fearlessness, more self-less and pro-social behaviors, some control over normally autonomic responses, and possibly some paranormal functionality. These improvements in normal human functioning provide some evidence that there are practices that develop these abilities, and as such we might want to consider adopting them to develop this capability. There are however insufficient studies of expert meditators and more research of adepts is called for that explores the relationship between levels of attentional skill and increases in functionality. We propose in search of the superhero, that if conventional mindfulness training can already augment mental and physical capabilities, a more serious inquiry and translation of its advanced methods into mainstream psychological theory is warranted.

## Introduction

Whilst the study of the behaviors and attributes of heroes has undergone a renaissance in recent years (Allison and Goethals, 2013), the characteristics of a superhero has remained the stuff of fiction. What we are gathering about our everyday heroes however is certainly inspiring and seemingly within our grasp: they are characterized by prosocial activity, though sometimes contrary to the status quo (Staats et al., 2008) they share some but not all of the qualities of moral role models such as a respect for humanity; they have a willingness to act in accord with their own ideals; and they often sacrifice self-interest for the sake of others (Colby and Damon, 1992).

Yet while all of us appear intermittently capable of such heroic acts, we don't seem to have the ability to be consistently heroic (Walker et al., 2010), let alone be relied upon to save the world (Becker, 1973). In fact our choices usually depend upon the situation (Zimbardo, 2007) as it seems our character is often too fragmented (Doris, 2002) to be reliable (Fischer et al., 2006). However, when we do act heroically such behavior seems to contain three core elements: service to others; is voluntarily chosen; and it brings possible risk or cost to the individual (Franco and Zimbardo, 2006, 2011). Typically people we see as heroes are known for demonstrating outside the norm behaviors (Hakanen, 1989), and perhaps we might question if we could do the same, or at least be able to develop these attributes over time. However not much is known on what might be predictable developmental steps (Erikson, 1982) that create a hero (Baltes et al., 2006; Callina et al., 2016) and whether we could be trained as children or adults to predictably think and behave heroically (Slagter et al., 2011; Jones, 2017).

To assist in this inquiry, Franco and Zimbardo (2006) proposed an approach that fosters “heroic imagination”—the cultivation of a heroic ideal that can “guide a person's behavior in times of trouble or moral uncertainty” (Franco and Zimbardo, 2006, p. 31). Since then Kohen et al. (2017) have made some headway by suggesting four trainable skills: developing an heroic imagination about how you would act; cultivating a sense of empathy and commonality with others; practicing habits of small-scale helping; and acquiring skills or traits specific to heroic actions. Other training approaches are beginning to emerge that focus on areas such as: developing an understanding of the bystander effect; challenging prejudice, group perception and stereotyping; improving situational awareness; and the development of greater understanding of how social conformity can reduce the likelihood of taking heroic actions (Franco et al., 2016). Yet on the whole there remains little research on hero training (Efthimiou, 2016b; Franco et al., 2016) and is a clear gap in the literature.

What could also be asked, in line with Aristotle's function argument that all things have a function and their meaning is found in the full expression of that function (Gomez-Lobo, 1988), is what is the full function or potential of a human being and could we learn to activate this. This points to a gap in the heroism literature—a discussion on human potential (Baltes and Staudinger, 2000; Vitterso, 2004) and whether our everyday hero, with training could push these frontiers even further and develop capacities akin to a superhero?

To explore this, and consistent with Allison (2016) recommendation that the science of heroism adopt a multidisciplinary approach and explore a range of diverse perspectives, we turn to a popular method of self-improvement—mindfulness, to see what it can offer to this discussion. What we find, buried in its origins of Buddhist psychology (Vago and Silbersweig, 2012), are claims that its master practitioners can in fact develop superhero-like capacities such as levitation, telekinesis, age-lessness and other extraordinary feats of superhuman ability. In an era where mindfulness is under the microscope, we ask if this ancient science of mental training can provide any scientific proof that a superhero could be possible. We define super-heroism as the demonstration of abilities well-beyond the expected norms of human capacity, including a control over mental and physical processes that can transcend the known laws of physics.

By way of introduction, we first cover off on the mental states that are proposed as necessary precursors to such abilities: the eight levels of concentration known as “jhana” states that map out progressively each stage of attention as mental control increases. We then investigate what abilities (“siddhis”) are proposed to arise as a natural outcome of such mental mastery. We then turn to the research and probe the evaluation of advanced mindfulness practitioners for any data that supports the development of such abilities.

As such we review literature that describe super-human capacities that supersede norms of the common day, and we explore whether there is any proof that the proposed practices develop these capacities normally reserved by superheroes. We argue that if indeed advanced practitioners can demonstrate at least some evidence of advanced functioning (Wilber, 2006), then if people wish to emulate our heroes/superheroes, they might want to consider adopting such practices to develop their capacities to achieve this goal.

### The Path of Mindfulness

Common to all major religious traditions is the claim that their most actualized practitioners operate at the level of superheroes, whilst still being fully human—that is with supernormal attributes (Radin, 2013). Like Buddhist psychology from which mindfulness originated, in the Hindu tradition dozens of such powers (known as siddhis) are seen to exist (Menon, 2007), as documented in Patanjali's Yoga Sutras (Honorton, 1981; Vivekananda, 2014). These abilities are viewed as natural milestones, with control over the external world seen as growing parallel with internal development (Maheshwarananda, 2004).

Yet whilst religions are littered with stories of great feats (Rama, 1999), and indeed living examples of individuals such as Amma in the Hindu tradition (Paramatmananda, 1987) or the Dalai Lama in Buddhism (Dalai Lama, 2002) are seen as miraculous within their traditions, such paths, unlike myths, offer techniques grounded in methodology that is repeatable and testable (Khyentse, 1993; Natarajan, 2000; Simao et al., 2016). Indeed science is now entering a dialogue with these traditions to understand and test the validity of these approaches (Ricard and Thuan, 2004; Braud, 2008).

However whilst the end point of Buddhist psychology is heightened meta-awareness leading progressively to enlightenment (Trungpa and Goleman, 2005), the more recent presentation of mindfulness is more concerned with subjective well-being (Teasdale et al., 2000) and is defined as non-judgmental attention to the present moment (Kabat-Zinn, 2011) and an ability to self-regulate one's behavior in a more prosocial way (Tacon et al., 2004). In contrast to enlightenment, the range of possible practice outcomes include present moment awareness (Cahn et al., 2013), attentional regulation (Vago and Nakamura, 2011) and loving kindness (Salzberg, 2011).

As such the current field or possibly “neo-mindfulness” does not yet have a cohesive or comprehensive model (Shear, 2006) that delineates a predictable stepwise progression of the practitioner that leads to the foundational state of non-dual awareness (Bharati, 1976) from which it is argued the supernormal qualities develop. In fact Fox et al. (2016) evaluation of functional neuroimaging studies of these different meditation methods found dissociable patterns of brain activation and deactivation for each style.

The critique of current mindfulness is that it has been taken from its origins and re-presented in a decontextualized manner that, whilst highly correlated with improved mental health, misses some of the richness or complexity offered by its parent philosophy (Kudesia and Nyima, 2015). This has caused construct validity issues as evidenced by diverging definitions of key terms, a range of practices (Siegel et al., 2008) that are hard to formalize as mindfulness (Shear, 2006) and somewhat diluted training methods (Teasdale et al., 2003).

Furthermore, there is a growing literature documenting significant differences between beginners and advanced mindfulness practitioners (long term meditators) who may be using the same “methods” but attaining very different “states.” One example is the transition from beginner to adept (from effort to effortless) where there is a decreased need for effort-based disengagement from distracting mental processes (Dunne, 2011), with that difference presenting in very different brain patterns.

One characteristic of the adept is their advanced states of non-dual awareness (Grof, 1972; Josipovic, 2014), also known as self-transcendent events (Yaden et al., 2017), anomalous experiences (Cardena et al., 2000), or even religious experiences (James, 1902). These terms all refer to a dissolution of the sense of an abiding and separate self or “anatta” (Pérez-Remón, 1980) and a perception of oneness or connectedness to people and surroundings. Such opening or awakening to the non-dual experience is seen to result from the removal of the cognitive, perceptual, and sensory layers of information processing leading to a more expanded and unitary state of consciousness (De Castro, 2015).

This deconstruction process is also described in Campbell (1949) depiction of the hero's journey (the saint in particular), which outlines the stages of human development, during which people undergo moral, mental, emotional, physical, and spiritual transformation. “The individual, through prolonged psychological disciplines, gives up completely all attachment to his personal limitations, idiosyncrasies, hopes and fears, no longer resists the self-annihilation that is prerequisite to rebirth in the realization of truth, and so becomes ripe, at last, for the great at-one-ment.” Campbell (1949), p. 236 Transpersonal theorists have subsequently introduced psychological nomenclature to better delineate this process of self-realization (Maslow, 1970; Prendergast, 2003). However, it is depicted it is only this advanced stage and its subsequent mental states that the emergence of the so called superpowers is seen to begin.

As mindfulness is one such methodology and its outcomes are now being empirically evaluated, it is well-positioned to answer questions about such advanced states and abilities. Yet whilst mindfulness is now being scientifically scrutinized, the possibility of special abilities as an outcome of practice, with a few exceptions, has received little attention (Anand et al., 1961; Benson et al., 1982; Roney and Solfvin, 2006). One of the challenges is that the scientific method or data gathering approach often inherent in research is based on the materialist or reductionist premise that if it cannot be verified it does not exist—“Of that which we can't speak about, we should remain silent” (Wittgenstein, 1922, p. 189).

Understandably the prospect of supernormal abilities, whilst about naturally occurring phenomena, is not currently easy to verify. Buddhist scholar Alan Wallace offers some assistance: “In Buddhism, these are not miracles in the sense of being supernatural events, any more than the discovery and amazing uses of lasers are miraculous—however they may appear to those ignorant of the nature and potentials of light. Such contemplatives claim to have realized the nature and potentials of consciousness far beyond anything known in contemporary science. What may appear supernatural to a scientist or a layperson may seem perfectly natural to an advanced contemplative…” (Wallace, 2007, p. 103).

Some guiding principles for a balanced study of the topic are offered by the Dalai Lama who firstly encourages scientific responsibility (see also Sedlmeier, 2011). “I am well-aware, however, of the danger of tying spiritual belief to any scientific system…Great vigilance must be maintained at all times when dealing in areas about which we do not have great understanding. This, of course, is where science can help.” He then goes on to also encourage open mindedness toward abilities achieved through advanced practice “After all, we consider things to be mysterious only when we do not understand them. Through mental training, we have developed techniques to do things which science cannot yet adequately explain” (Dalai Lama, 2002, p. 230–243).

### Buddhist Psychology and Special Abilities

Originally Gautama Buddha is said to have achieved the state of perfect enlightenment as an outcome of meditation and as a result he is described as having access to supernormal abilities (Olson, 2017). He developed the four noble truths (life is empty of inherent satisfaction; the source of suffering is craving; suffering ceases when craving ends and awakening to one's true nature occurs; and there is a method to achieve this (which includes mindfulness), as a summary of the required deconstruction process (Anderson, 1999). In the later Tibetan Buddhist school of Zchogchen this same sequence is followed with the first step of training (“Treckcho”) targeting non-dual awareness followed by the advanced practice of “Togal” which actually includes training in special abilities in service of others (Sogyal, 1992). This order is seen as critical (most traditions advise against the pursuit of abilities for their own sake) lest the practitioner develop and use abilities derived from concentration that are not pure, ego-less and in service of humanity (narcissistic personality disorder)—aka the antihero (Wilber, 2006; Jones, 2017).

The Buddhist path has produced different approaches since its origin however all forms; the Theravada, Mahayana and Vajrayana, assert the possible perfection of the practitioner, known, respectively, as the arahant, bodhisattva, and mahasiddha (Katz, 1990). In these advanced or final stages of practice it is claimed that practitioners develop special abilities in comparison to normative functioning. These aptitudes are seen as a natural and necessary outcome of seeing through the illusion that the subjective self and objective phenomena are separate and inherently existent entities (Nirbhay et al., 2015). In early Theravadin texts six abilities known as the “abhinnas” (also known as siddhis) or higher knowledge are reported as progressively attainable by practitioners who have typically passed the fourth (of eight) levels of concentration known as “jhana” states.

The levels of jhana are an account of progression up the ladder of mental control. The Buddha in a number of sutras exhorts his disciples to develop the jhana states, and the first four figure in the training of right concentration in the 8-fold path (Gunaratana, 1988). The average mindfulness practitioner would rarely enter the first level of mental control depicted by these states (Clough, 2011) hence empirical studies seldom evaluate such practitioners. However, as they are seen as the mental foundation for the subsequent unfoldment of the superpowers in Buddhist theory (Lodro, 1998) we note how they present.

In brief, first jhana (joy) is the state of continuous concentration with no interruptions and pleasant sensations (bliss) in the background. In second jhana (contentment) one lets go of the previous physical and emotional pleasure and moves to motionless, quiet contentment. Third jhana (utter peacefulness) is a sense of equanimity with no positive or negative feeling and an all pervading, peaceful one-pointedness of mind. In fourth jhana (infinity of space) there is the experience of absorption without form, attention shifts beyond the body as if watching oneself, and the self is experienced as the expanse of empty space (Narada, 1980).

In most accounts, the practitioner must have progressed past the first four “material” jhanas before extra mental abilities start to manifest (Tsong Khapa, 2002), however there is some divergence as to when they are seen to manifest. The remaining four jhanas during which such abilities are seen to develop are: fifth jhana (infinity of consciousness)—awareness that infinite space includes one's own consciousness and attention shifts to infinite consciousness (oneness with nature and existence); sixth jhana (no-thingness)—realization that infinite consciousness itself is empty of inherent existence and that all is impermanent and changing; seventh jhana (neither perception nor non-perception)—going beyond the duality of perception nor non-perception and yet still aware; eighth jhana (cessation)—cessation of overt consciousness with only subtle perception remaining (can appear unconscious) yet perfectly one with everything.

Once, as the result of long term training, a practitioner has achieved_the preliminary states, in Buddhist theory it is predicted that six categories of abilities (De Silva, 2005) can arise [in Hinduism they can number as high as twenty four; (Maheshwarananda, 1990)]. These wide-ranging supernormal (Radin, 2013) faculties are: (1) Performing Miracles (psychokinesis)—the attainment of extra-ordinary physical powers including disappearing, walking on water, passing through solid objects and flying; (2) Celestial hearing (clairaudience): the ability to hear sounds from far away, even other realms; (3) Knowledge of thoughts (telepathy)—can communicate without words and understand unspoken languages including animals; (4) Knowledge of past and future (knowledge beyond time)—can know events from the past and future of both themselves and others including previous and future life cycles; (5) Celestial vision (clairvoyance)—the ability to see things in minute detail or far away, can see through solid objects, can see in the dark or the nature of someone's mind, vision is free and unobstructed; (6) Eradication of all defilements (end of suffering)—the realization of enlightenment or nibbāṇa (the achievement of most value), the practitioner has now transcended the cycle of birth and death (Jacobsen, 2012).

As comprehensive and extraordinary as this complete set of capacities is, it is thought that some of the simpler faculties can occasionally occur naturally in some people with no or moderate training (the usual scope of western parapsychological research) or whilst progressing along the path of mindfulness training (Roney-Dougal, 2006). However, the full complement of attributes are typically seen to be reached only after having achieved the highest state of concentration, and are under the complete control of the practitioner (Thrangu, 2001). What is normalizing, amidst such extraordinary descriptions, not unlike the notion of the banality of heroism, is that such attributes are seen in Buddhist theory as a natural expression of human capacity.

### Mindfulness Research

Both the above described concentration states and supernormal faculties are well-beyond the experience of the average person and are more representative of a superhero than our typical hero. And as mindfulness practices and results have now attracted significant empirical investigation it is reasonable to ask what evidence has been found that these practices deliver any preliminary indicators of such outcomes. If there are some signs of their existence this will expand our current understanding of both human potential and just how heroic can we become.

Two issues we face however are the scarcity of highly advanced mindfulness practitioners available for testing (Ricard, 2011) and scarcity of data as most researchers currently focus on normal range of processing (Sedlmeier et al., 2012). In view of these limitations (this current inquiry is a call for more research of advanced practitioners) we will investigate what documented results are available from mindfulness training. We do this by examining the following categories: health and resistance to disease, anti-aging, pro-social behavior, consciousness, control of autonomic responses, and the paranormal. As such we are looking for any evidence, across a range of categories, that mindfulness practice increases human functioning beyond normal expectations.

### Health and Resistance to Disease

In terms of the basic fundamentals of health, reviews of mindfulness meditation interventions (Chen et al., 2012) have found them in some cases to be as effective as conventional antidepressants in the treatment of depressive and anxious disorders. For example Pascoe et al. (2017) found that meditation can decrease physical correlates such as cortisol, blood pressure, resting heart rate, blood glucose, and cholesterol. Such results suggest a modulation of the sympathetic nervous system and hypothalamic pituitary adrenal system and lend support to such practices triggering a physiologically adaptive response to psychological stress (Nesse et al., 2016).

In terms of disease prevention, whilst mental stress suppresses the immune response, regular meditation has been associated with an increased resistance to disease (Ventriglio et al., 2015). In support of this meditation has been negatively associated with neurodegenerative conditions, such as Alzheimer's (Huang et al., 2016), reduces the expression of pro-inflammatory genes in blood cells (Creswell et al., 2012), and has been found to trigger structural brain changes in patients with Parkinson's disease (Pickut et al., 2013).

García-Campayo et al. (2018) found an unexpected epigenetic response to long term meditation practice suggesting a molecular response to mindfulness practice involving crucial regulators related to a range of common diseases. This ties into other findings in epigenetic research such as the identification of “KIFAP3 (Kinesin-associated protein 3) which prolongs the lifespan of motor neuron disease sufferers (dubbed the “hero gene”). As Efthimiou (2016a) questions “By tapping into the hero epigenome and its regenerative properties it may be possible to conceive of the development of not only more effective life strategies at a psychosocialspiritual level, but chemical or natural compounds at the medical level…” (p. 3). This raises the question as to whether the quality of one's mental state could act as a prophylactic against ill health and even play some role in altering the genetic makeup of an individual. Whilst this is longitudinal change, in keeping with evolutionary theory, it supports the notion that altering one's mental state can change one's physical state.

### Anti-aging

Reduced attentional processes have often been reported in the elderly, and it has been proposed that deterioration of the governing mechanism common to other cognitive domains such as memory could explain age-related decline (Gazzaley and D'esposito, 2007). To see if mindfulness practices could ameliorate such aging effects, Sperduti et al. (2016) compared older adults with long-term meditation experience and age-matched older adults with no meditation experience and young adult novices. Cognitive decline of the age-matched control was not observed in the meditators, and older meditators' performance was indistinguishable from the young control (replicated by Chiesa and Serretti, 2010).

In studies looking at processing speed and aging, Hawkes et al. (2014) reported that reaction time differences between meditation practitioners and controls remained significant even after controlling for age. Similar studies showed a reduced attentional blink in middle-aged meditators, compared with an aged matched control group (van Leeuwen et al., 2009).

Some explanation of this could be found in research by Luders et al. (2015) who found that age-related gray matter atrophy in these structures was consistently reduced in meditators. Similarly Lazar et al. (2005) also found that meditation was responsible for slowing age-related thinning of the frontal cortex. A possible mechanism for this is that mindfulness practices may recruit frontal brain regions responsible for attentional control, boost neuronal plasticity in these structures (Sperduti et al., 2016) and offer some immunity against the effect of aging and the deterioration of cognitive processes. Such findings are relevant to this inquiry as the implication is that mindfulness practices can partially reverse the effect of time and actually alter the physical structure of the brain.

### Cognitive Performance

Mindfulness has been linked to improved cognitive performance in specific neural networks (Cahn and Polich, 2006; Malinowski, 2013; Tang et al., 2015). For example in an EEG study van Lutterveld et al. (2017) reported that the alpha band functional network was better integrated in experienced meditators than in novice meditators during meditation. This suggests a more efficient information exchange between different brain areas (Xue et al., 2014).

Similarly when Tanaka et al. (2015) compared first time meditators with long term meditators, the beginners exhibited higher beta in the frontal cortex during attentional tasks than their counterparts (more concentrated effort), suggesting that experienced meditators maintain attention with less exertion than normals. In Lomasa et al. (2015) EEG studies they found a significant increase in both alpha and theta activity during meditation which suggested a state of relaxed alertness conducive to adaptive functioning. Using neuroimaging, Lazar et al. (2005) found meditators had increased thickness indicative of improvement in function in the cortical regions specifically related to emotional, cognitive, and sensory processing.

Such encephalography and neuroimaging findings suggest that mindfulness training can increase the speed, versatility and neural structures of its practitioners. With such confirmation it is reasonable to ask just how fast and flexible could such processing become as mastery developed. Could we like Neo in his superhero training in the Matrix ask the same question: “What are you trying to tell me, that I can dodge bullets?” (Wachowski et al., 1999).

### Resilience and Fearlessness

Event-related brain potential (ERP) research of Sobolewski et al. (2011) found that meditation practitioners are less affected by stimuli with adverse emotional load and show an attenuated brain response to viewing negative pictures. Such effects may be mediated by both a reduced negative affective response and an increased positive emotional attitude (positive affect) toward self and others (Wadlinger and Isaacowitz, 2011; Reva et al., 2014)—an outcome goal of mindfulness practices.

This is in line with the transactional model of stress and coping (Lazarus and Folkman, 1984) where resilient individuals initiate a secondary response of decentring from the stressor so as to also attend to their response rather than just the aversive stimulus (Garland et al., 2009). With meditation practice this ability has been found to be more developed and in advanced practitioners can become an automatic and effortless emotional regulation strategy, which in turn reduces the activation of sympathetic nervous system processes such as cardiovascular arousal (Pavlov et al., 2015). Studies comparing experienced meditators to controls and short-term meditators have demonstrated a physiological profile suggestive of an alert, but hypometabolic state in which there is decreased sympathetic nervous system activity, and yet also increased parasympathetic activity (Young and Taylor, 1998; Jha et al., 2007).

Research into neural structures has found greater gray matter concentration in the hippocampus of advanced meditators, suggesting that practice over time enhances circuitry for extinction learning and retention (Holzel et al., 2008; Luders et al., 2013). Similarly thickness of the medial prefrontal cortex has also been found to be directly correlated with extinction retention after fear conditioning. That is, increase in size following extensive training might structurally explain how meditators modulate fear better in that they have more evolved hardware or capacity (better, faster, stronger) in terms of survival response to threat (Ott et al., 2011).

Garland and Howard (2013), building on Kabat-Zinn et al. (1985) seminal work, reported a mindfulness intervention that reduced attentional bias for pain-related stimuli in patients in chronic pain. Through the decoupling of cognitive and sensory faculties associated with the perception of pain, practitioners were able to better perceive painful stimuli without a mental response (Rosenzweig et al., 2010). A physiological correlate of this is the finding that experienced meditators also have a more rapid decrease in skin conductance following a painful stimulus (Goleman and Schwartz, 1976) and a decreased startle amplitude (Levenson et al., 2012). As fearlessness and heroism are correlated, this finding is also relevant to our inquiry as expert mindfulness practitioners, have a more adaptive fear response and appear better positioned to act heroically in threatening settings.

### Pro-social Behavior

Heroically-relevant behaviors such as sacrificing needs in service of others, acts of loving kindness and ethical behavior (Shaner et al., 2017) are increasingly being examined in the fields of cognitive science (Boyer, 2003) and neuroscience (Newberg and Iversen, 2003). Sometimes known as the “moral emotions” (Yaden et al., 2017), they are the proposed outcome of the Mahayana Buddhist teaching—the way of the “bodhisattva” (translated as enlightened hero) or enlightenment for the benefit of all sentient beings (Khyentse, 1993). The practices are divided into compassion (“karuna”) which seeks to reduce the suffering of others, and loving-kindness (“maitri”) which desires to increase the happiness of others (Fredrickson et al., 2008).

This altruistic dimension of mindfulness is now well-documented (Neff, 2011; Salzberg, 2011), with a meta-analysis of the prosocial effects of all meditation methods combined finding that there was an increase in prosocial behavior (Kreplin et al., 2018). Repeated evaluation of the Transcendental Meditation techniques (Mahesh Yogi, 1995) consistently reported decreased levels of aggression and violence (Haegelin et al., 1999). An investigation of specific loving kindness meditation methods (the cultivation of unrestricted readiness and intention to help others) by Lutz et al. (2008) found that practitioners showed more emotional reactivity to sounds of people in distress and a stronger insula response (linked to interoception and empathy).

Neurological studies of loving kindness have found an increase in frontal-parietal gamma coherence and power, and self-reported clarity amongst long term practitioners (Lutz et al., 2004). Again researchers have reported changes in gray matter concentration (Holzel et al., 2011), with changes in brain regions involved in emotion regulation, empathy, and perspective taking (Fan et al., 2011), and functional increases in the insular cortex during compassion exercises (Manna et al., 2010; Desbordes et al., 2012). Essentially the fundamental principle of neuroplasticity is borne out here—if it is stimulated it increases.

The cultivation of compassion through loving-kindness practices is viewed as a critical component of mindfulness as it builds ethical qualities seen as a necessary balance for a method that increases mental power. As such (Ricard, 2009) cautions using mindfulness training on its own “Bare attention, as consummate as it might be, is no more than a tool …which can also be used to cause immense suffering. Obviously what is entirely missing is the ethical dimension of a mindfulness that deserves the qualification of ‘wholesome’ and can lead to enlightenment.” In fact if we are exploring all the possible features of a superhero, the attributes of altruism and non-violent tendencies, whilst not supernormal capacities, are probably the distinguishing qualities that separate them from the villain.

### Consciousness

As we can now identify which parts of the brain are affected during meditation practice (Demertzi et al., 2013) methods like single pointed concentration (e.g., focusing on the breath) have been correlated with decreases in activity in areas like the lateral pre-frontal cortex (PCC) and parietal cortex (Posner and Rothbart, 2009; Tang et al., 2009). Garrison et al. (2013) neuro-feedback analyses have also revealed that the mental states derived from such methods such as “undistracted awareness” or “effortless doing” correspond to pre-cingulate cortex deactivation, whereas other mental states such as “distracted awareness” or “control” correspond to PCC activation.

In terms of consciousness Shapiro (2008) found that decreased activity in the pre-frontal cortex (Brewer et al., 2013), which occurs in long-term meditators, can also lead to a disassembling of the processes responsible for our normal experience of time and space (Berkovich-Ohana et al., 2013). As the insula operates the integration between interoceptive and exteroceptive signals (Critchley et al., 2004), it promotes the generation of the emotional states that so often define our sense of self (Cauda et al., 2011; Seth et al., 2012). Ananthaswamy (2014) identified that as the insula has been found to check body states every 125 ms to create a series of emotional frames of reference, any interruption to this such as meditation (altered states of consciousness have been shown to have enhanced activity in the anterior insula) interferes with this otherwise robust sense of self.

In fact Andrews-Hanna et al. (2010) identified eleven brain centers involved in “selfing” of different types. For example they found the dorsal medial prefrontal cortex subsystem is preferentially activated for the “self-and-other,” the medial temporal lobe system manages the “self-in-time” and the core network of two centers is involved in all activities. If the prefrontal cortex subsystem is deactivated, one loses a sense of time, and feels timeless, and if the temporal lobe is deactivated, an individual feels a sense of unity or no separation. In terms of the responsiveness of these systems to meditation, both the medial prefrontal cortex and posterior cingulate cortex have been found to be deactivated in meditators relative to controls Brewer et al. (2011), giving further evidence of the plasticity and physiological correlates of consciousness (Travis and Pearson, 2000).

Farrer and Frith (2002) found that the parietal regions are responsible for the brain's capacity to sense bodily boundaries or one's position in space and hence make distinctions between self and non-self. When advanced meditators attain a state of thought-free emptiness there is decreased activity in areas responsible for body sense such as the medial parietal areas (Hinterberger et al., 2014). Similarly, when there is a sense of expanded space, selflessness and timelessness, activity is significantly reduced in the areas involved in perceptual processing (Berkovich-Ohana et al., 2013). Such neurological changes seem to correspond with the fourth jhana state in the Buddhist typology (infinity of space) where there is the experience of formless absorption and the self is perceived as the expanse of empty space.

In respect of the experience of “no self” when advanced meditators move from effort to effortlessness and attain a “no thought” state, the cessation of intentional control is observable with a reduction in overall brain activity (Johnstone et al., 2012) and is marked with decreases in the areas responsible for body sense (medial parietal areas). Several studies have also found that long-term meditation is associated with increased theta synchronization resulting in blissful experiences (Vaitl et al., 2005; Hagerty et al., 2013) that possibly correspond to the first jhana state. This is consistent with other research that theta activity, peaking in the left prefrontal region, correlates with emotionally positive experiences (Aftanas and Golocheikine, 2001). In terms of neuroimaging, meditation appears to increase the size of regions key to meta-awareness and introspection (rostrolateral prefrontal cortex) and body awareness (sensory and insular cortices), while decreasing the size of regions related to the default mode network (Buckner et al., 2008; Fox et al., 2014).

Such data suggests that the practice of meditation can cause both structural and functional alterations within the neural networks that typically promote and maintain consciousness (Lehmann et al., 2012). The implications of are that long-term meditation practitioners who have proficiency in meditative techniques can experience “the mind observing itself” (Dalai Lama et al., 1991), and as a result of reduced identification with the mind-body signals, respond to events in literally a “self-less” way. Furthermore, the data is in line with the descriptions of the early jhana states presented in Buddhist psychology which are preliminary to the evolution of special abilities commensurate with our superhero.

### Control of Autonomic Responses

In early research Green and Green (1977) assessed under laboratory conditions the capacity to control autonomic responses. In an initial trial Swami Rama an expert meditator with 30 years' experience increased the temperature difference between the left and right sides of his hand after 3 min to 11F apart (neural controls over the radial and ulnar arteries in the wrist are located within a few millimeters of each other in the central nervous system). In a second experiment on demand the subject stopped his heart from pumping blood for 16 s, with no detectable pulse (other than an atrial flutter on the EKG) before returning heart rate back to baseline with no ill effects.

Benson et al. (1982) studied subjects practicing the Tibetan Buddhist meditational practice g-tummo (heat) and found they had the capacity to increase the temperature of their fingers and toes (a prophylactic to frostbite) by as much as 8.3°C. In a more recent follow up study of this phenomenon, Kozhevnikov et al. (2013) reported reliable increases in axillary temperature from the normal range (37.5°C) up to 38.3°C among g-tummo meditators accompanied by increases in alpha, beta, and gamma power. More recently Kox et al. (2014) report not only similar results with the Hoff method but also found that participants had increases in the release of epinephrine which enhanced disease protection.

It has also been found that with subjects in the deepest state of concentration (samadhi), the automatic regulatory process of breathing can be overridden and the breathing rate can drop to two or three breaths a minute (Lazar et al., 2000; Austin, 2006), well-below the average of about fifteen times per minute. This was first demonstrated again by Swami Rama who brought his conscious breathing down to 1–2 breaths per minute (Green and Green, 1977). A more recent application of the utility of this was the heroic Thailand cave rescue of school students, where the teacher (an ex monk) taught the boys breathing meditation methods to both relax them and reduce oxygen intake to optimize the chance of survival.

What is unusual about the above research results is that not only are homeostatic mechanisms normally controlled by the central nervous system, but in the case of the g-tummo temperature regulation findings, the detectors of heat and effectors for changing temperature are located in the extremities (e.g., the hands and feet) and are not set up as a reflex mechanism to be overridden by cognitive commands. Such unexplainable evidence that the mind can have direct influence over physical mechanisms normally outside our control is a possible indicator of the capability for telekinesis (remote control of physical systems) in the Buddhist system of supernormal abilities.

### Paranormal

In early research Braud (1974) introduced the concept of the “psi-conducive state” as a relaxed non-distracted mental state which is supportive of the perception of non-tangible phenomena (Dukhan and Rao, 1973). Later research (Honorton, 1977) built on this by investigating whether in fact meditation acted as a psi-conducive state (Harding and Thalbourne, 1981; Roll and Zill, 1981; Schmeidler, 1994) with still interesting but mixed results (most studies used beginner/intermediate meditators not advanced practitioners or ‘adepts’).

In more recent research Roney and Solfvin (2006) explored whether long-term meditation practice facilitates psi awareness by evaluating Indian practitioners with three levels of training: students (0.3–15 years practice); sannyasins (1–10 years practice); and swamis (4–33 years practice). In preliminary studies the advanced group (swamis) scored significantly better than both the students and sannyasins however this was not achieved in the full study. In a follow up study, it was hypothesized that “years of meditation” of Tibetan Buddhist meditators would correlate positively with psi scoring (advanced meditators would correctly choose hidden targets more than beginners). Roney-Dougal et al. (2008) findings confirmed that overall scoring (accuracy) was significantly correlated with years of practice.

Rao (1994) conducted tests to compare subliminal perception scores (SP) of novices and transcendental meditation (TM) practitioners. A comparison revealed that the TM groups performed significantly better than the control group on the SP task leading the authors to propose that the contributing factor was the reduced distractibility/sensory noise reduction achieved by greater mindfulness levels. In an earlier study exploring the relationship between meditation and extra sensory perception (ESP), Rao et al. (Rao, 1984) carried out three series of forced-choice ESP tests and one free-response test to see whether subjects would obtain higher scores after meditation. The results showed that participants obtained significantly higher ESP scores in tests immediately after meditation than at pre-test (immediately before meditation).

In another experiment by Green and Green (1977) expert meditator Swami Rama was assessed under laboratory conditions to mentally move an object without exerting any physical force. Wearing a face mask and sitting six feet away to prevent any effects of airflow, Rama recited a mantra, and after a loud exclamation and a word of command, the needle rotated ten degrees toward him. This was replicated twice in the presence of six medical doctors and experimental scientists.

The human potential movement, has taken a multidisciplinary approach to such abilities by using the theories of biological evolution and modern physics to explain the supernormal abilities derived from meditation (Ronson, 2005). Kripal (2011) proposes the paradigm “evolutionary mysticism” as a theoretical framework to interpret these abilities in scientific terms. The broad range of abilities under their scrutiny include such things as the works of geniuses like Brahms who reported first seeing his compositions in their finished form (Abell, 1964) or Indian mathematician Srinivasa Ramanujan, who with little formal training made significant contributions to pure mathematics through his perception of Namagiri writing equations in his dreams (Schwartz, 2010).

Whilst such special abilities were omitted from this inquiry as they are not a direct result of mindfulness practices, they are still part of a large compendium of studies that meet scientific criteria and present the case for a range of extraordinary abilities (Radin, 1997). It is of note however, that whilst such natural abilities are genuinely intriguing and worthy of further investigation, they are not necessarily correlated with a more enlightened or altruistic character (Sogyal, 1992). Furthermore, Roney and Solfvin (2006) identified that abilities that are the consequence of systematic meditative practice were more consistent than those that were spontaneous and untrained. This last finding lends further support to the need to investigate reliable training methods that develop such abilities.

## Discussion

In exploring the existence of special abilities corresponding with that of the superhero, the field of mindfulness is in a unique position, through its theoretical rigor and empirical investigation to extract, from its traditional religious context, both cohesive theory and verifiable techniques. However, as Sogyal (1992) says, to be truly scientific it must be “shorn of dogma, fundamentalism, exclusivity, complex metaphysics, and culturally exotic paraphernalia” (p. 151). This investigation sought to separate out fact from fiction and see if there is any evidence for the presence of special abilities as a result of long term mindfulness practice. That is to identify if there is a link between advanced mindfulness practices and the development of functionalities commensurate with a superhero. The inquiry did in fact find some evidence of heightened levels of functioning as a result of exposure to such practices.

By way of summary at the level of health this included: predictable decreases in important indicators such as cortisol, blood pressure, heart rate and cholesterol (Pascoe and Bauer, 2015; Pascoe et al., 2017), negative associations with mindfulness and neurodegenerative conditions (Huang et al., 2016), evidence that mindfulness acts as a prophylactic to ill health; and some kind of epigenetic response to mindfulness practice (García-Campayo et al., 2018). In terms of cognitive functioning we found mindfulness practice to assist in the maintenance of reaction time, working memory and cognitive flexibility during aging (Sperduti et al., 2016); increased thickness in the cortical regions related to emotional, cognitive, and sensory processing (Lazar et al., 2005); and a slowing of the reduction in age-related attentional performance and gray matter (Pagnoni and Cekic, 2007). Advanced mindfulness practitioners through metacognitive awareness and decentring were also found to be less affected by adverse stimuli (Garland et al., 2009); have improved emotion regulation and fear response (Etkin et al., 2011); decreased sympathetic nervous system activity and increased parasympathetic activity (Benson, 2000); and have lower pain sensitivity with higher pain thresholds (Grant et al., 2010).

Finally mindfulness training was also found to be associated with: increases in regions responsible for meta-awareness and introspection (Buckner et al., 2008; Fox et al., 2014); a sense of expanded space, selflessness and timelessness (Berkovich-Ohana et al., 2013); increases in pro-social behavior such as loving-kindness and compassion (Salzberg, 2011); and changes in brain regions involved in self-empathy and perspective taking (Holzel et al., 2011). There was evidence that some expert practitioners had the capacity to control normally involuntary processes such as: body temperature (Kox et al., 2014) and heart rate (Green and Green, 1977); significantly reduce their breathing rate (Lazar et al., 2000; Austin, 2006); and demonstrate a relationship between meditation and both extra sensory perception (Rao, 1984; Roney-Dougal et al., 2008) and telekinesis (Green and Green, 1977).

Such findings go a long way to indicate that advanced practitioners demonstrate abilities beyond the expected norms of human functioning and some data even suggests abilities we might expect from our superheroes. What such information points to is that it may be possible for an ordinary person, with the correct training to start on the path of superhero; an ego-less individual who progressively unlocks his or her higher capacities in service of mankind. This could also be relevant for people across a broad range of disciplines or organizational settings: educators, therapists, clergy, and those in leadership (even politicians).

In evaluating the range of mindfulness studies, one issue that arose was the absence of baselines for experienced meditators. An example of this is van Leeuwen et al. (2009) who compared a group of “expert meditators” and cited a practice range between 1 and 29 years. Such instances were not uncommon and demonstrate limited understanding of the effects of different levels of training. For example Hauswald et al. (2015) found an unexpected correlation between high gamma source power (effort) and high Mindful Attention Awareness Scale (MAAS) scores. The effects however were mainly carried by meditators who could definitely concentrate and had reported high MAAS scores but had not actually reached expert level (effortless attention), as evidenced by the EEG which revealed they were still exerting effort. This highlights the need to have more “length of practice” demarcations in mindfulness studies and suggests that highly advanced practitioners may have a superior level of functioning (effortless) that standardized self-report mindfulness measures can't always distinguish. In such cases the use of self-report measures may benefit from being coupled with an EEG to better map neuroscientific differences in advanced practitioners.

In view of the ambiguity of what constitutes expert practitioners (years or hours are not sufficient indicators) what is called for are more studies that separate out the levels of concentration attained by practitioners (possibly mapped against something like the eight jhana states proposed in Buddhist psychology). Once concentration levels are more clearly delineated clinical, neuroscientific and paranormal testing of these practitioners would give us a clearer picture of how impactful mindfulness training was in the development of heroic/super heroic potential. One obstacle to this is that research focuses on the normal range of processing rather than the extraordinary or supernormal (Kasamatsu and Hirai, 1973; Radin, 2013) partially because of the difficulty in getting “elite” meditators to participate in research. As monk and molecular biologist Ricard (2011) highlighted, many highly accomplished monks are contemplative hermits disinterested in displaying their faculties, which makes finding sufficient cohorts at the top end of functioning somewhat challenging.

Along with the above recommendation we also suggest that in regards to the study of human functioning, the dominant materialist theory of mind undergo further evaluation (Barber, 1961; Lehmann et al., 2001). The materialist position toward consciousness is that mind is only the result of physiological processes; that each person's mind is a discrete and separate entity with communication only possible through the physiological senses; and that consciousness is limited to the time/space continuum (Schwartz, 2010). However, like the burgeoning field of mindfulness there is also a growing amount of theory, empirical observation, and now neuroscience research (Luo and Niki, 2003) that questions the edges of this model (Hastings, 2002).

For example, Frecska and Luna (2006) proposed a neuro-ontological interpretation of non-physical experiences that considers information processing, when overloaded, as not needing to be limited to hierarchically organized and interconnected neural networks. Rather beyond the neuroaxonal level, they posit, an interface can emerge where there is a transition from neurochemical to quantum physical events (quantum entanglement). The implication of this is that some states of consciousness could have quantum origin and hence not be limited by signal locality (wired vs. wireless connection) and hence not easily measurable by current instruments.

Interestingly such an information processing theory fits a theoretical framework that could support at least three of the six non-local abilities outlined in Buddhist psychology (clairaudience, clairvoyance, and telepathy). In the coming years as the research of quantum mechanics, neuroscience and mindfulness increasingly integrate (Davis and Vago, 2013) there may be a greater theoretical and empirical meeting that makes provision for some of the supernormal faculties.

Mindfulness theory and practice, as derived from Buddhist psychology, is unapologetically positive about our human potential and the current inquiry offers the reader, or budding hero/ superhero, some pointers that might advance him or herself on “the superhero's journey.” However, further investigation into this dimension will contribute to heroism science theory and the need for such an integration of eastern and western knowledge in psychology is echoed by Sedlmeier et al. (2012): “we believe every effort should be made to extract precise psychological theories that are relevant for meditation from both the Hindu and Buddhist approaches” (p. 1162).

Meanwhile what has been found is an encouraging indicator of increased human capability due to regular mindfulness practice. As these preliminary results were derived from practitioners who generally only mastered the introductory states of concentration there may be still richer data awaiting us. Like all technological leaps previously seen as impossible, we may indeed need to expand the profile of the everyday hero to include functionality previously reserved for super heroes.

## Author Contributions

PJ is the sole contributor to the article.

### Conflict of Interest Statement

The author declares that the research was conducted in the absence of any commercial or financial relationships that could be construed as a potential conflict of interest.
